# Glycated Serum Protein Genetics and Pleiotropy with Cardiometabolic Risk Factors

**DOI:** 10.1155/2019/2310235

**Published:** 2019-04-04

**Authors:** Matthew P. Johnson, Ryan Keyho, Nicholas B. Blackburn, Sandra Laston, Satish Kumar, Juan Peralta, Suman S. Thapa, Bradford Towne, Janardan Subedi, John Blangero, Sarah Williams-Blangero

**Affiliations:** ^1^South Texas Diabetes and Obesity Institute, School of Medicine, University of Texas Rio Grande Valley, Brownsville, Texas 78520, USA; ^2^Department of Human Genetics, School of Medicine, University of Texas Rio Grande Valley, Brownsville, Texas 78520, USA; ^3^The University of Texas at Austin, Austin, Texas 78705, USA; ^4^Menzies Institute for Medical Research, University of Tasmania, Hobart 7000, Australia; ^5^Tilganga Institute of Ophthalmology, Gaushala, Bagmati Bridge, P.O. Box 561, Kathmandu, Nepal; ^6^Department of Population Health and Public Health Sciences, Boonshoft School of Medicine, Wright State University, Kettering, Ohio 45435, USA; ^7^Department of Sociology and Gerontology, College of Arts and Science, Miami University, Oxford, Ohio 45056, USA

## Abstract

Measurements of fasting glucose (FG) or glycated hemoglobin A1c (HbA1c) are two clinically approved approaches commonly used to determine glycemia, both of which are influenced by genetic factors. Obtaining accurate measurements of FG or HbA1c is not without its challenges, though. Measuring glycated serum protein (GSP) offers an alternative approach for assessing glycemia. The aim of this study was to estimate the heritability of GSP and GSP expressed as a percentage of total serum albumin (%GA) using a variance component approach and localize genomic regions (QTLs) that harbor genes likely to influence GSP and %GA trait variation in a large extended multigenerational pedigree from Jiri, Nepal (*n* = 1,800). We also performed quantitative bivariate analyses to assess the relationship between GSP or %GA and several cardiometabolic traits. Additive genetic effects significantly influence variation in GSP and %GA levels (*p* values: 1.15 × 10^−5^ and 3.39 × 10^−5^, respectively). We localized a significant (LOD score = 3.18) and novel GSP QTL on chromosome 11q, which has been previously linked to type 2 diabetes. Two common (MAF > 0.4) SNPs within the chromosome 11 QTL were associated with GSP (adjusted *p*value < 5.87 × 10^−5^): an intronic variant (rs10790184) in the *DSCAML1* gene and a 3′UTR variant (rs8258) in the *CEP164* gene. Significant positive correlations were observed between GSP or %GA and blood pressure, and lipid traits (*p* values: 0.0062 to 1.78 × 10^−9^). A significant negative correlation was observed between %GA and HDL cholesterol (*p* = 1.12 × 10^−5^). GSP is influenced by genetic factors and can be used to assess glycemia and diabetes risk. Thus, GSP measurements can facilitate glycemic studies when accurate FG and/or HbA1c measurements are difficult to obtain. GSP can also be measured from frozen blood (serum) samples, which allows the prospect of retrospective glycemic studies using archived samples.

## 1. Introduction

Glycemic level influences a variety of medical conditions. Hyperglycemia is a condition that is the result of excess circulating glucose in the blood. While quantitative definitions of hyperglycemia vary, the American Diabetes Association considers fasting glucose (FG) of 100-125 mg/dL or glycated hemoglobin A1c (HbA1c) of 5.7-6.4% as prediabetic and FG greater than 125 mg/dL or HbA1c greater than 6.4% as diabetic [1]. Diabetes is a condition that affects millions worldwide, and the global prevalence continues to increase, especially in low- to middle-income countries [2]. Diabetes causes numerous health conditions, exacerbates existing health conditions, and is a risk factor for blindness, kidney failure, heart attacks, and other cardiovascular disease events. Diabetes-related complications significantly contribute to global mortality rates [3].

Standardized clinical assays are in place to measure glycemia, and these tests are used to diagnose diabetes and monitor glycemic control over time. These assays include the measurement of FG (plasma [FPG] or serum [FSG]) and HbA1c. FG is a cross-sectional measurement of glycemic levels at that time point while HbA1c is a measure of long-term glycemic control over an 8- to 12-week period based on the time it takes to form glycation of hemoglobin as well as the lifespan of a red blood cell. However, these assays are not without their limitations. FG may be impractical in situations where individuals must travel a significant distance prior to sampling, making a fasting state difficult to achieve. A meaningful HbA1c test is dependent on the absence of blood disorders such as anemia, which can generate spurious results [1].

Glycated serum proteins (GSP) are proteins within the body that have undergone glycation and circulate in the blood. The large majority (90%) of GSP consists of glycated albumin (GA) [4], a globular protein in plasma whose main purpose is to regulate the oncotic pressure of the blood. Due to the shorter half-life of serum proteins, compared to red blood cells, GSP levels show glycemic control over a period of 2 to 3 weeks [5]. Used in conjunction with measures of HbA1c, GSP measures can monitor glycemic control over a 2- to 8-week period via the calculation of a “glycation gap” [6]. In addition to monitoring short-term glycemic control, GSP is reported to be associated with the risk of atherosclerosis [7, 8], microvascular complications of diabetes [9], and cardiovascular disease-related outcomes [10]. Assessments of GSP do not require sampled individuals to be fasting and can be performed using frozen samples [11].

FG and HbA1c are heritable traits [12], and genetic variants have been shown to influence variation in FG [13, 14] and HbA1c [15], respectively. The goals of this study were (1) to determine whether observed variation in GSP and GSP expressed as a percentage of total serum albumin (%GA) is influenced by genetic factors (i.e., heritability), (2) to determine whether GSP and %GA are correlated with cardiometabolic risk factors (i.e., pleiotropy), and (3) to localize areas of the genome that harbor positional candidate genes likely to influence GSP and %GA trait variation (i.e., genetic linkage). The genetics of GSP and %GA and the relationships of these measures with cardiometabolic traits were assessed in a large family-based cohort from rural Nepal.

## 2. Materials and Methods

### 2.1. Study Population

The Jirel Family Studies began in 1987 and focused on the Jirel population of eastern Nepal. Approximately 2,500 members of the Jirel population have participated in prior studies including anthropological investigations [16, 17], assessments of population structure [18, 19], genetic epidemiology studies of susceptibility to parasitic worm infections [20, 21], genetic studies of growth and development [22, 23], and investigations of the genetics of ocular disease [24]. The long running research conducted in the region has resulted in the collection of extensive genealogical information on the Jirel people. All individuals who have previously participated in research studies belong to a single extended multigenerational pedigree, which makes this cohort an extremely powerful resource for genetic studies [25]. The data and samples used for this study are part of an ongoing project investigating the genetic epidemiology of ocular traits and ocular disease [24]. All procedures were conducted in accordance with ethical standards and were approved by the University of Texas Rio Grande Valley Institutional Review Board and the Nepal Health Research Council. Informed consent was obtained from all individuals participating in the study.

### 2.2. Phenotype Data Collection

#### 2.2.1. Cardiometabolic Trait Measurements

1. *Blood Pressure.* A single systolic blood pressure (SBP) and diastolic blood pressure (DBP) reading was recorded from individuals in a seated position using the Welch Allyn Connex ProBP digital blood pressure device (Welch Allyn Inc., Skaneateles Falls, NY, USA).
2. *Body Mass Index (BMI).* Height was measured using a mobile stadiometer (Seca, Chino, CA, USA), and weight was measured using an analog weight scale (Seca). BMI was calculated as weight in kilograms divided by the square of height in meters.
3. *Lipid Panel.* Total cholesterol (TC), high-density lipoprotein cholesterol (HDLC), and low-density lipoprotein cholesterol (LDLC) concentrations were determined from nonfasting serum samples using the ACE® Cholesterol, HDLC, and LDLC reagent packages, respectively, and run on the ACE Axcel® Clinical Chemistry System (Alfa Wassermann Diagnostic Technologies, LLC., West Caldwell, NJ, USA) in the South Texas Diabetes and Obesity Institute (STDOI) phenotyping laboratory, Brownsville, Texas. Briefly, a sample volume of 3 *μ*L for each of the TC, HDLC, and LDLC assays was used and assay parameters such as assay-specific reagent volumes, incubation times, reaction wavelengths, and bichromatic correction wavelengths were preset on the ACE Axcel® instrument by the manufacturer.

#### 2.2.2. GSP and %GA Measurements

GSP concentrations were determined from nonfasting serum samples using the Diazyme Glycated Serum Protein assay kit (Diazyme Laboratories Inc., Poway, CA, USA) and run on the ACE Axcel® Clinical Chemistry System (Alfa Wassermann) in the STDOI phenotyping laboratory, Brownsville, Texas. Briefly, a sample volume of 10 *μ*L was added to 200 *μ*L of Diazyme's Reagent 1 (containing proteinase K) and incubated at 37°C for 5 minutes to digest the GSP into low molecular weight glycated protein fragments (GPF). Following this incubation, 50 *μ*L of Diazyme's Reagent 2 (containing fructosaminase) was added to the GPF solution to catalyze the oxidative degradation of GPF to yield protein fragments or amino acids, glucosone, and hydrogen peroxide (H_2_O_2_). The released H_2_O_2_ is measured as a colorimetric end-point reaction, and absorbance between 546 nm and 600 nm is proportional to the concentration of glycated serum proteins. A two-point calibration step was also performed, in duplicate, at readings of 0 *μ*mol/L (blank) and 485 *μ*mol/L. The Diazyme GSP assay exhibits a linear range of 21.0 to 1,354.0 *μ*mol/L and has no significant interference from ascorbic acid, bilirubin, conjugated bilirubin, glucose, hemoglobin, triglycerides, or uric acid [26].

To calculate %GA (i.e., the amount of glycated serum albumin expressed as a percentage of the total circulating serum albumin), we also measured total serum albumin with the ACE® Albumin Reagent package run on the ACE Axcel® Clinical Chemistry System (Alfa Wassermann). Briefly, a sample volume of 3 *μ*L was used and serum albumin assay parameters such as assay reagent volume, reaction wavelength, and bichromatic correction wavelength was preset on the ACE Axcel® instrument by the manufacturer. GSP values (*μ*mol/L) were converted to %GA by applying the following equation recommended by Diazyme [26]:

(1) $$\begin{array}{c} \%\text{GA}=\frac{\text{GSP}\left(\mu \text{mol}/\text{L}\right)\times 0.182+1.97}{\text{total serum albumin}\left(\text{g}/\text{dL}\right)}+2.9. \end{array}$$

### 2.3. Genome-Wide Genotypes

Jirel DNA samples were genotyped using Illumina's Human660W-Quad v1 BeadChip (Illumina Inc., San Diego, CA, USA) containing ~550,000 SNP loci. A total of 200 ng of genomic DNA for each sample was processed according to Illumina's Infinium HD Assay Ultra protocol. BeadChips were imaged on Illumina's iScan System with iScan Control Software (v.3.2.45). Normalization of raw image intensity data, genotype clustering, and individual sample genotype calls was performed using Illumina's GenomeStudio software (v2010.2), Genotyping Module (v1.7.4). Illumina's predefined genotype cluster boundaries were used to denote SNP genotype cluster positions (Human660W-Quad_v1_H.egt). Genotype assay quality control measures were assessed with Illumina's internal assay performance metrics.

### 2.4. Statistical Methods

#### 2.4.1. Heritability Estimates

To estimate the heritability of measured traits including GSP and %GA, we used a variance component approach as implemented in SOLAR [27]. Here, we estimate the narrow sense heritability,

(2) $$\begin{array}{c} h^{2}=\frac{\sigma_{\text{a}}^{2}}{\left(\sigma_{\text{a}}^{2}+\sigma_{\text{e}}^{2}\right)}, \end{array}$$

by partitioning the observed phenotypic variance (*σ*_p_^2^) into its additive genetic and environmental components. In its most simplistic form, the observed covariance matrix of a quantitative trait in a pedigree of arbitrary size (*n*) is modeled as

(3) $$\begin{array}{c} \Omega =\left(2\Phi \times \sigma_{\text{a}}^{2}\right)+\left(I_{\text{n}}\times \sigma_{\text{e}}^{2}\right), \end{array}$$

where Ω is the *n* × *n* covariance matrix, 2Φ is the *n* × *n* coefficient of relationship structuring matrix, *σ*_a_^2^ is the variance in the observed trait due to additive genetic effects, *I*_n_ is the *n* × *n* identity structuring matrix for an implied individual-specific environmental component, and *σ*_e_^2^ is the variance in the observed trait due to random (unmeasured) individual-specific environmental effects.

Age, age^2^, sex and their interactions, and BMI were included as covariates in the additive genetic models. Variation in serum storage conditions (time, temperature) may impact protein glycation [11, 28]. Therefore, serum storage time, defined as the number of days in ultralow temperature (<-80°C) storage from the sample collection date until the assay date, was included as an additional covariate. For any traits that exhibited a departure from a normal distribution, an inverse normal transformation was applied to correct these distribution errors before reanalyzing the trait(s).

#### 2.4.2. Quantitative Bivariate Analysis

Using the kinship coefficients among family members, the correlation between two traits can be partitioned into its additive genetic and random environmental components [29]. Therefore, the magnitude of the genetic covariance (i.e., pleiotropy) between the glycated serum protein traits and cardiometabolic traits (blood pressure, BMI, and lipid panel) was assessed by employing a bivariate quantitative genetic analysis procedure as implemented in SOLAR. This procedure quantifies the overall relationship between the two tested traits (phenotypic correlation (*ρ*_p_)) by evaluating the magnitude of both the additive genetic (*ρ*_g_) and random environmental (*ρ*_e_) correlations where

(4) $$\begin{array}{c} \rho_{\text{p}}=\left(\rho_{\text{g}}\times \sqrt{h_{\text{A}}^{2}\times h_{\text{B}}^{2}}\right)+\left(\rho_{\text{e}}\times \sqrt{\left(1-h_{\text{A}}^{2}\right)\times \left(1-h_{\text{B}}^{2}\right)}\right), \end{array}$$

where *h*_A_^2^ and *h*_B_^2^ denote heritability estimates for traits “A” and “B,” respectively. In this framework, the likelihood of models that constrain the additive genetic (*ρ*_g_) correlation between the traits (A and B) to zero is compared to the likelihood of a model that allows for the additive genetic correlation between the traits to be estimated. These pleiotropy analyses highlight whether two traits are influenced by a common set of genes (*ρ*_g_ = −1 or 1; complete pleiotropy), overlapping but nonidentical sets of genes (−1 < *ρ*_g_ < 0 or 0 < *ρ*_g_ < 1; incomplete pleiotropy), or unique sets of genes (*ρ*_g_ = 0; no pleiotropy).

#### 2.4.3. Genotype Cleaning

PREST-plus v4.09 [30] was used to confirm known pedigree relationships and identify possible sample swap errors. Genotype-based sex checks and variant filtering to include autosomal variants only and a 95% call rate per person were performed using PLINK v1.90b3m [31]. A total of 479,686 clean autosomal SNPs were available for the analysis.

#### 2.4.4. Genome-Wide Linkage Analysis

Genotype array data was analyzed with IBDLD v3.33 [32], using methodology previously described [33], to generate chromosome-wide empirical kinship estimates and to calculate multipoint estimates of identity-by-descent (MIBD) at 1 cM intervals across autosomal chromosomes. Using SOLAR, empirical kinship estimates and MIBDs were employed in a variance component approach to conduct a genome-wide linkage scan of GSP and %GA to identify genomic regions (quantitative trait loci (QTLs)) harboring genes that influence the variation observed in these glycated serum protein metrics. Our genetic linkage analyses were conducted in a subset of Jirel individuals with genome-wide genotype data available (*n* = 1,087). Age, age^2^, sex and their interactions, and BMI and serum storage time were included as covariates in our linkage analysis models.

#### 2.4.5. Measured Genotype Association Analysis

The classical measured genotype approach for association analyses [34, 35], as implemented in SOLAR [36], was used to analyze variant genotype data within the 1-LOD (95% confidence) interval of significant QTL signal(s). To ensure robust statistical testing, autosomal SNPs with five or more observed copies were prioritized for QTL-specific association analyses (471,074 SNPs out of a total of 479, 686 clean SNPs). Similar to our linkage analyses, pedigree-based kinships were used for our association analyses, and age, age^2^, sex and their interactions, and BMI and serum storage time were included as covariates in our measured genotyped association models.

## 3. Results

Phenotypic data were available from 1,800 individuals (55% female) from the single extended multigenerational Jirel pedigree. The mean (SD, range) age is 42.4 (16.6, 18 to 88) years, and a summary of the cardiometabolic traits is presented in Table 1.

### 3.1. Glycemic Trait Heritability Estimates

In the Jirel pedigree, the heritability estimates of both GSP (*h*^2^ = 0.159, *p* = 1.15 × 10^−5^, SE = 0.044) and %GA (*h*^2^ = 0.152, *p* = 3.39 × 10^−5^, SE = 0.044) were significant, indicating that variation in GSP and %GA is influenced by additive genetic factors in this population.

### 3.2. Glycemic Trait Pleiotropy with Cardiometabolic Risk Factors

We performed a quantitative bivariate analysis to assess the direction and strength of the phenotypic correlation between GSP or %GA and cardiometabolic traits in the Jirel pedigree (Table 2). The most significant result was a positive correlation between GSP and TC (*ρ*_p_ = 0.144, *p* = 1.78 × 10^−9^). SBP exhibited a significant positive correlation with both GSP (*ρ*_p_ = 0.089, *p* = 2.08 × 10^−4^) and %GA (*ρ*_p_ = 0.066, *p* = 0.0062). Other significant (*p* < 0.01) positive correlations were observed for GSP with DBP and LDLC. The only significant (*p* = 1.12 × 10^−5^) negative correlation was observed for %GA with HDLC. There was evidence to suggest that common genetic loci, in addition to unique genetic loci, may influence the variation observed in GSP and TC in the Jirel pedigree (*ρ*_g_ = 0.351, *p* = 0.025).

### 3.3. Glycemic Trait Linkage and Association

**Figure 1:**
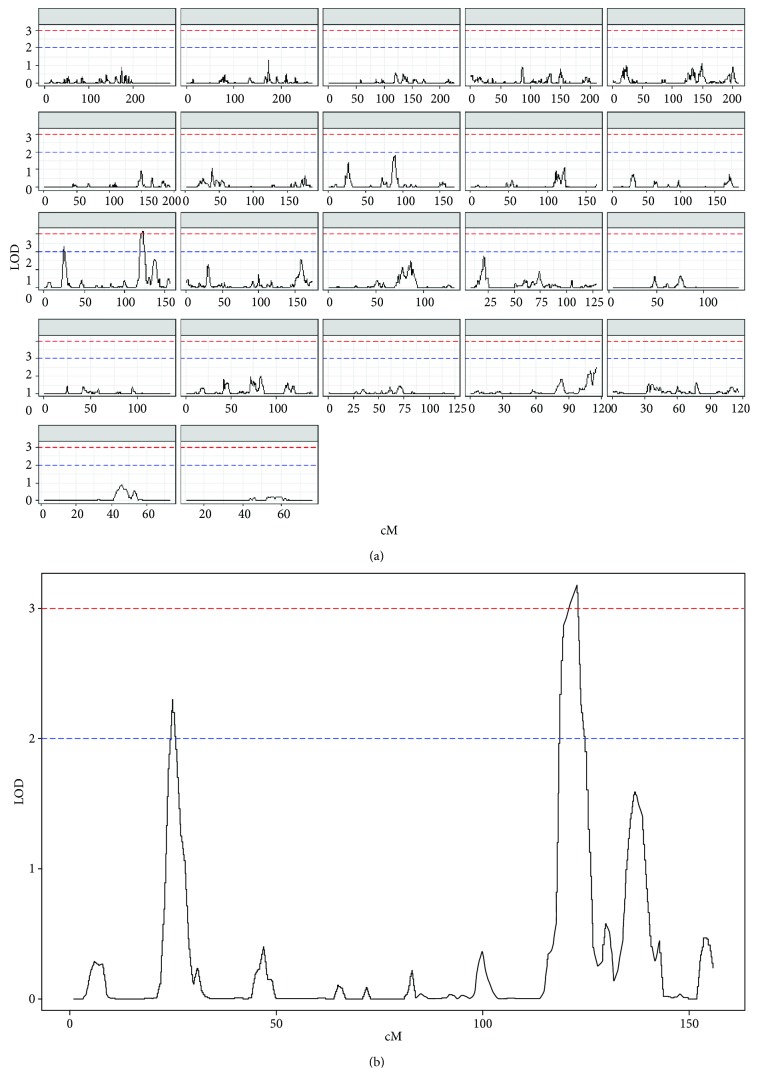
Genome-wide linkage (a) and chromosome-specific linkage (b) plots for the significant GSP QTL on chromosome 11. Dashed red line: significant linkage (LOD ≥ 3.0). Dashed blue line: suggestive linkage (2.0 ≤ LOD < 3.0).

For GSP, we identified a significant QTL (LOD = 3.18) on chromosome 11 at 123 cM (119,235,404 bp) (Figure 1). For %GA, we identified a suggestive QTL (LOD = 2.01) on chromosome 4 at 197 cM (186,584,255 bp).

**Figure 2:**
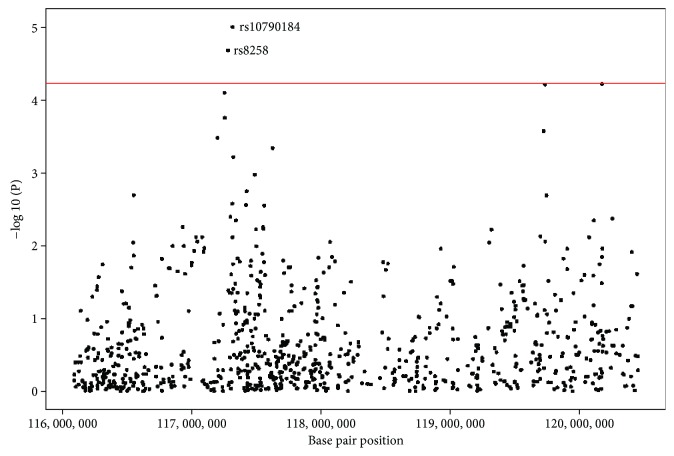
Measured genotype association plot for the GSP chromosome 11 QTL 1-LOD (95% confidence) interval. Solid red line: QTL-specific Bonferroni-corrected significance criterion (-log10(*P*) ≥ 4.23). Genomic coordinates: GRCh37/hg19.

To interrogate our significant GSP QTL further, we performed a measured genotype association analysis for all SNPs within the 1-LOD (95% confidence) interval (118-125 cM; 116,094,471-120,456,605 bp). Two SNPs satisfied our QTL-specific Bonferroni-corrected significance criterion (*p* < 5.87 × 10^−5^; 852 SNPs with 5 or more copies of the rarer variant) (Figure 2). The first SNP (rs10790184; *p* = 1.00 × 10^−5^; *β* = 0.202) is an intronic variant in the *DSCAML1* (DS cell adhesion molecule like 1) gene, and the second SNP (rs8258; *p* = 2.10 × 10^−5^; *β* = 0.194) is a 3′UTR variant in the *CEP164* (centrosomal protein 164) gene. The rs10790184 (MAF = 0.478) and rs8258 (MAF = 0.408) SNPs are both common and explain approximately 1.86% and 1.78% of GSP variation in the Jirel pedigree, respectively.

## 4. Discussion

In this study, we set out to assess the genetics of GSP and %GA, which are possible alternative or complementary measures of glycemia. Standard practice to measure blood sugar levels is to test FG and/or HbA1c but not GSP or %GA. Feasibly, GSP or %GA could be used for historical samples or in situations where measuring FG is impracticable (environmental conditions may complicate maintaining a 12-fast) or when HbA1c results are spurious due to conditions (e.g., anemia) that may be unknown to both the patient (research participant) and the clinician (researcher).

To test the genetics of GSP and %GA, we utilized a cohort from rural Nepal (the Jirel population). All individuals of the Jirel population belong to a single extended multigenerational pedigree which makes this cohort an extremely powerful resource for genetic studies [25, 37]. We observe that GSP and %GA levels are significantly heritable and additive genetic factors account for approximately 16% and 15% of the total phenotypic variation, respectively. Our significant GSP heritability estimate is, however, in contrast to a study of nondiabetic monozygotic (MZ) and dizygotic (DZ) twins, which did not support genetic factors influencing GSP variability [38]. These contrasting results may be due to the power of the two samples and differences in the overall genetic structure of the two family-based cohorts. A greater number of higher degree relationships (as there are in the Jirel pedigree) are likely to further minimize the confounding of shared environmental signals with genetic signals. Other factors that may explain the different results between our study and the twin study by Cohen et al. include age- and sex-specific differences, as well as the ethnic diversity between the two study populations. The average age of our study is 42.4 years compared to 54.0 and 49.9 years for the MZ and DZ twin samples, respectively; our study included both males and females compared to the female-only twin study; and the Jirels of Nepal are of South Asian origin whereas the female twins were likely of Caucasian ancestry.

Perturbations in cardiometabolic trait homeostasis are key risk factors in numerous disease outcomes, for example, hyperglycemia and diabetes [39], dyslipidemia and coronary heart disease [40], and hypertension and stroke [41]. Collectively, measures of these cardiometabolic traits that exceed clinical thresholds constitute metabolic syndrome. Therefore, the direction of our observed phenotypic correlations between the glycated serum protein traits and lipid traits (positive correlation with TC and LDLC, negative correlation with HDLC) and blood pressure traits (positive correlation) is a likely observation, especially in individuals at a high risk of metabolic syndrome or any other cardiovascular disease-related event. However, our glycated serum protein-cardiometabolic trait correlation data are not in full agreement with a small number of other studies that have tested similar correlations [7, 8, 42]. It is difficult to deduce whether this discordance is real or artefactual given the limited number of studies that have investigated correlations between glycated serum protein traits and cardiometabolic traits at this stage.

Our result demonstrating significant linkage for GSP is of considerable interest. Our linkage region (11q23.3) sits within an area on chromosome 11q that has been previously linked to type 2 diabetes (T2D). The chromosome 11qter region was initially linked to T2D in a cohort of Pima Indians [43] and has subsequently been replicated in two independent cohorts of Caucasian families [44, 45] and a cohort of Mexican American families [46]. Additionally, there are several promising positional candidate genes within the GSP QTL that lend further support to our finding. The *TREH* (trehalase) gene encodes an enzyme that hydrolyses trehalose, a disaccharide formed from two glucose molecules, and the activity of this enzyme in plasma has been found to be higher in diabetic patients compared to nondiabetic patients [47]. In the *ARHGEF12* (Rho guanine nucleotide exchange factor 12) gene, a functional SNP (rs148969251) was identified to associate with insulin sensitivity in nondiabetic patients [48]. The rs148969251 SNP, however, was not present on the Illumina Human660W-Quad v1 BeadChip. Several other promising positional candidate genes include *C2CD2L* (C2CD2 like), a gene whose function has been shown to regulate insulin secretion from beta cells [49, 50]; *C1QTNF5* (C1q and TNF related 5), a gene suggested to have a role in the development of T2D [51]; and *BACE1* (beta-secretase 1), a gene that has been shown to have a role in glucose homoeostasis in a mouse knockout model [52]. How these genes and/or genetic variants may impact levels of GSP and/or regulation of serum protein glycation remains to be determined.

A recent genome-wide association (GWA) study supports the role of additive genetic factors to influence GSP variation [53]. Here, two genome-wide significant loci were associated with serum fructosamine in white and black cohorts of unrelated individuals, respectively. The serum fructosamine GWA signal in the black population was for an intergenic SNP (rs2438321) on chromosome 11 at 98,500,410 bp [53]. The distance between the GWA signal identified by Loomis et al. [53] and our GSP QTL is approximately 17.6 megabases and therefore likely represents two independent signals.

We acknowledge that our study is not without its limitations. The single blood pressure measurements are potentially inflated: “white-coat hypertension.” An average of multiple measurements would be a better sampling strategy and would provide a more robust measure. Also, we are unaware of any diseases (e.g., hyper- and hypothyroidism) that may perturb normal albumin metabolism, which may impact the accurate measurement of glycated serum protein levels.

## 5. Conclusion

We have demonstrated that observed variation in glycated serum protein is significantly influenced by additive genetic factors and identify a novel QTL for this glycemic biomarker. The glycated serum protein QTL overlaps with an area of the q-arm of chromosome 11 that has previously been linked to T2D, and several positional candidate genes in this region have been shown to regulate insulin sensitivity and secretion. We also show glycated serum protein traits are correlated with other cardiometabolic traits, which suggests these measures of short-term glycemic control are a novel biomarker for dyslipidemia and hypertension; however, additional studies are warranted to confirm or refute this possibility. Measuring glycated serum proteins can also be conducted from frozen blood (serum), which facilitates retrospective glycemic studies utilizing archived samples.

## Figures and Tables

**Table 1:** Descriptive statistics for cardiometabolic traits in the Jirel pedigree.

	Blood pressure		Anthropometry	Lipid traits			Glycemic traits	
	SBP (mm Hg)	DBP (mm Hg)	BMI (kg/m^2^)	TC (mg/dL)	HDLC (mg/dL)	LDLC (mg/dL)	GSP (*μ*mol/L)	%GA (%)
Mean	132.1	84.0	22.3	176.7	51.8	103.3	353.5	18.5
SD	20.9	10.7	3.7	49.9	18.3	38.7	92.0	4.7
Min.	89	52	14.1	47.0	8.0	17.0	70.5	6.1
Max.	248	140	38.8	409.0	141.0	294.0	797.2	49.1

SD: standard deviation; Min: minimum; Max: maximum.

**Table 2:** Phenotypic and genetic correlations between the glycated serum protein metrics and cardiometabolic traits measured in the Jirel pedigree.

Trait pairs	Phenotypic correlation		Genetic correlation^∗^	
	*ρ*_p_ (SE)	*p* value	*ρ*_g_ (SE)	*p* value
GSP-SBP	0.089 (0.024)	2.08 × 10^−4^	-0.027 (0.166)	0.872
GSP-DBP	0.067 (0.024)	0.0056	-0.053 (0.161)	0.742
GSP-BMI	0.047 (0.024)	0.052	0.017 (0.122)	0.891
GSP-TC	0.144 (0.024)	1.78 × 10^−9^	0.351 (0.149)	0.025
GSP-HDLC	-0.019 (0.024)	0.424	-0.177 (0.161)	0.273
GSP-LDLC	0.096 (0.024)	8.05 × 10^−5^	0.263 (0.136)	0.057
%GA-SBP	0.066 (0.024)	0.0062	-0.069 (0.177)	0.689
%GA-DBP	0.020 (0.024)	0.402	-0.140 (0.172)	0.407
%GA-BMI	0.006 (0.024)	0.796	-0.002 (0.128)	0.988
%GA-TC	0.035 (0.024)	0.148	0.289 (0.163)	0.077
%GA-HDLC	-0.106 (0.024)	1.12 × 10^−5^	-0.238 (0.166)	0.162
%GA-LDLC	-0.021 (0.024)	0.382	0.072 (0.146)	0.623

SE: standard error. ^∗^Additive genetic heritability estimates (*h*^2^: 0.149 to 0.536) for all cardiometabolic traits were significant (*p* values: 1.15 × 10^−4^ to 1.14 × 10^−42^).

## Data Availability

The data used to support the findings of this study are available from the corresponding author upon request.
